# Prediction of avian influenza A binding preference to human receptor using conformational analysis of receptor bound to hemagglutinin

**DOI:** 10.1186/1471-2164-10-S3-S24

**Published:** 2009-12-03

**Authors:** Nipa Jongkon, Wanwimon Mokmak, Daungmanee Chuakheaw, Philip J Shaw, Sissades Tongsima, Chak Sangma

**Affiliations:** 1 Cheminformatics Research Unit, Department of Chemistry, Faculty of Science, Kasetsart University, Thailand; 2 National Center for Genetic Engineering and Biotechnology, National Science and Technology Development Agency, 113 Thailand Science Park, Phahonyothin Road, Klong 1, Klongluang, Pathumthani 12120, Thailand

## Abstract

**Background:**

It is known that the highly pathogenic avian influenza A virus H5N1 binds strongly and with high specificity to the avian-type receptor by its hemagglutinin surface protein. This specificity is normally a barrier to viral transmission from birds to humans. However, strains may emerge with mutated hemagglutinin, potentially changing the receptor binding preference from avian to human-type. This hypothesis has been proven correct, since viral isolates from Vietnam and Thailand have been found which have increased selectivity toward the human cell receptor. The change in binding preference is due to mutation, which can be computationally modelled. The aim of this study is to further explore whether computational simulation could be used as a prediction tool for host type selectivity in emerging variants.

**Results:**

Molecular dynamics simulation was employed to study the interactions between receptor models and hemagglutinin proteins from H5N1 strains A/Duck/Singapore/3/97, mutated A/Duck/Singapore/3/97 (Q222L, G224S, Q222L/G224S), A/Thailand/1(KAN-1)/2004, and mutated A/Thailand/1(KAN-1)/2004 (L129V/A134V). The avian receptor was represented by Siaα(2,3)Gal substructure and human receptor by Siaα(2,6)Gal. The glycoside binding conformation was monitored throughout the simulations since high selectivity toward a particular host occurs when the sialoside bound with the near-optimized conformation.

**Conclusion:**

The simulation results showed all hemagglutinin proteins used the same set of amino acid residues to bind with the glycoside; however, some mutations alter linkage preferences. Preference toward human-type receptors is associated with a positive torsion angle, while avian-type receptor preference is associated with a negative torsion angle. According to the conformation analysis of the bound receptors, we could predict the relative selectivity in accordance with *in vitro *experimental data when disaccharides receptor analogs were used.

## Background

Avian influenza H5N1 virus uses its hemagglutinin (HA) protein to bind with a host receptor before entering the cell. This protein binds avidly to the avian-type receptor. However, a major health concern is that HA mutation could alter the binding preference to that of human receptor, which could occur before the virus is completely adapted to its new host [1]. The incidences of human infection by H5N1 virus and the spectrum of H5N1 mutations are increasing [2-4]. Some of the mutated viruses could potentially infect humans and be spread person-to-person causing an outbreak [5,6].

**Figure 1:**
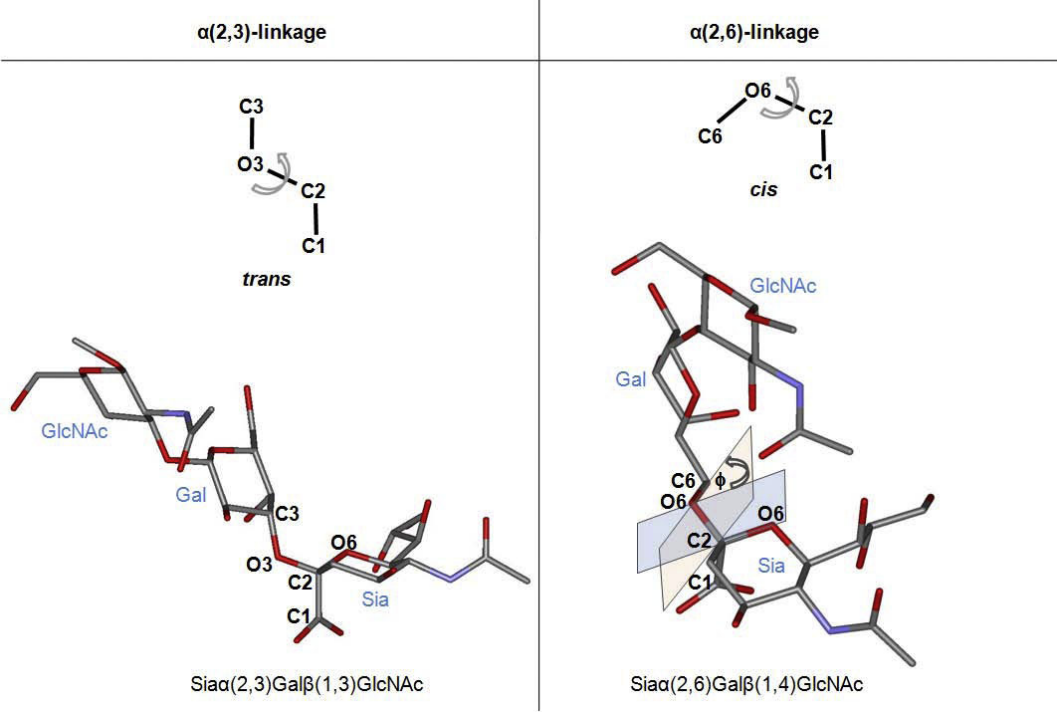
**Receptor analog structures used in the molecular dynamics simulations**. The left panel presents the defined torsion angle (Φ) of O6-C2-O3-C3 in the α(2,3) systems. The right panel presents the defined torsion angle of O6-C2-O6-C6 in the α(2,6) systems.

The host cell selectivity of influenza A viruses is mediated by the interaction of particular viral HA variants to different host cell receptor types. The cell receptor that is bound by HA is a penta-saccharide chain. The first sugar unit is sialic acid (Sia), followed by galactose (Gal), N-acetylglucosamine (GlcNAc), Gal, and glucose (Glc) units. However, the available X-ray crystal structures of the host cell receptor are in the tri-saccharide form as shown in Figure 1, precluding accurate simulation of the full-length receptor. Two types of receptor are bound by influenza virus: the first type has the α(2,3)-linkage between the first two units to form Siaα(2,3)Gal glycosides. The other receptor type contains an α(2,6)-linkage to form Siaα(2,6)Gal. In avian viruses, the preferred HA receptors are of the Siaα(2,3)Gal type, while most human viruses interact with Siaα(2,6)Gal glycoside receptors. Normally, the avian influenza virus H5N1 infects birds rather than humans or other mammalian hosts because their HA binds better to the avian-type Siaα(2,3)Gal glycoside receptor [7,8].

Despite the number of HA variants that have been reported in the protein database [9,10], we do not know enough about the binding mechanism to predict which HA variants can bind efficiently to the human receptor. The most reliable source of information to study the binding mechanism is from X-ray crystal structure. However, only a small number of H5 HA-receptor co-crystals are available in public databases [5]. Another approach to study which HA variants can bind preferentially to human receptor is by binding assay experiments which systematically screen interactions between HA variants and receptor analogs [11,12]. Nevertheless, to produce and screen many proteins in order to search for human-receptor binding HAs is impractical. Alternatively, insight into the binding mechanism can be observed by HA protein-receptor simulation to predict the binding potential between different HA variants and human receptor. By this means, one can effectively screen and assign priority to a small number of HA variants for further *in vitro *experiments.

Surveillance of H5 HA mutations over a number of years has led to the discovery of viruses with changes in receptor binding selectivity caused by mutation of the receptor binding site. The H5 HA X-ray structure from A/Duck/Singapore/3/97 (abbreviated as Sing-97) shows preferential binding to Siaα(2,3)Gal receptor, owing to Q222L and G224S mutations [5]. A Sing-97 descendent that infected a human in 2004, A/Thailand/1(KAN-1)/2004 (abbreviated as Kan-1), has a single mutation in the HA binding pocket (S129L). This variant, however, still binds preferentially with the avian receptor [13]. A Kan-1 derived strain with further HA mutations L129V and A134V is considered a quasi-species which exhibited higher selectivity toward human cell type receptor [14].

It should be noted that the mutated amino acids considered in the study are not the main binding residues and the mutations do not greatly alter the resulting complex structure. However, some of these mutations do indeed change the receptor type selectivity by contributing to changes in binding mechanism. This modification in host type preference could be caused by differential binding of mutually exclusive conformations of the cell receptor in different HA binding environments. It is hypothesized, that by comparing the conformations of different receptor types bound to different HA binding sites with known binding selectivity, we could distinguish different bound conformations. A recent study by Xu and coworkers [15] has shown that receptor binding preference of different influenza viral types can be modelled by simulation measuring receptor torsion angles. In this study, it is shown how the information from the available structures of HA complexed with avian and human receptors can be used to predict the HA binding selectivity from different HA variants in receptor-based conformational analysis by measuring a single torsion angle during Molecular Dynamic simulation. Furthermore, the predictions of receptor preference agree with the available in vitro binding data.

## Materials and methods

### Crystallographic datasets and HA variants used for simulation

X-ray crystallographic datasets of HA variants complexed with tri-saccharide receptor analogs were obtained from the PDB (Table 1). In the 1JSO dataset, the electron density is ill-defined for the Galβ(1,4)GlcNAc sugar residues. Therefore, to allow accurate comparisons between the different crystallographic templates in MD simulations, the structures of the equivalent sugar residues from the receptor in the 1RVZ structural template [16], the closest receptor structure available, were inserted into the 1JSO template. All glycosides were terminated with a methoxy group and were used as the input for molecular dynamics simulations. To prepare HA variants for MD simulation for which no crystallographic data are available, homology modelling was performed by three-dimensional alignment with the most similar structural template (1JSN and 1JSO for Sing-97 and Kan-1 variants; 1RVX and 1RVZ for A/Puerto Rico/8/34 (abbreviated as Puerto-34) variants) using the SWISS-MODEL server [17].

**Table 1:** Torsion angles of selected glycosides from protein data bank (PDB).

PDB code	HA Type	Ligand Type	Sugar Units	Torsion (Φ)	Resolution (Å)	Citation
1JSI	A/Swine/Hong Kong/9/98:H9	Siaα(2,6)Gal	Sia-Gal-GlcNAc-Gal-Glc	56.2	2.4	[5]
1JSO	A/Duck/Singapore/3/97:H5	Siaα(2,6)Gal	Sia	-	2.4	[5]
1RVT	A/Swine/Iowa/15/30:H1	Siaα(2,6)Gal	Sia-Gal-GlcNAc-Gal-Glc	69.7	2.4	[16]
1RVZ	A/Puerto Rico/8/34:H1	Siaα(2,6)Gal	Sia-Gal-GlcNAc	67.6	2.25	[16]
1JSN	A/Duck/Singapore/3/97:H5	Siaα(2,3)Gal	Sia-Gal-GlcNAc	-55.1	2.4	[5]
1RVX	*A/Puerto Rico/8/34:H1*	*Siaα(2,3)Gal*	*Sia-Gal-GlcNAc*	*-59.1*	*2.2*	[16]

A number of HA strains of known host selectivity were used to test whether Siaα(2,3)Gal would bind to HA with avian receptor preference in *trans*, and whether Siaα(2,6)Gal would bind to HA with human receptor preference in *cis*. The MD simulations on seven known HA variants with avian and human receptor types were performed: Sing-97, Puerto-34, Kan-1, mutated Kan-1, and three mutated Sing-97 systems. Among these systems, according to the available binding assay data, two of them, Sing-97 and Kan-1, bind Siaα(2,3)Gal stronger than Siaα(2,6)Gal [18,19]. One of the mutated Sing-97 strains (Q222L/G224S) is known to bind Siaα(2,6)Gal stronger, suggesting that these mutations could be important for changing the receptor preference from avian to human. The other strains except Puerto-34 can equally bind to both types of the receptor. For Puerto-34 there are no experimental data from binding assay, but we assumed strong human receptor binding for this is the strain since it caused a human pandemic [16].

Our prediction scheme was tested on two sets of data. The first set comprised four Kan-1 HA variants each with single mutations M226T, K189G, K218S, and L190P obtained as quasi-species from the source where Kan-1 was found [14]. The reason for testing these variants was to determine whether these strains are potentially harmful, *i.e. *stronger binding to human receptor than Kan-1. The second set contained three mutated Puerto-34 HA variants, Q222L, G224S, and Q222L/G224S (H5 numbering). These variants were chosen to determine what effect these mutations had on a different HA type (H1) to Sing-97 (H5).

### Molecular dynamic simulations

To permit comparison with previous experimental results [19], all the simulations were done using the same Glycam04 [20] parameters for receptor and AMBER 2003 force field for protein. Initial structure was first solvated using the TIP5P water model [21] in the truncated octahedron box. Energy minimization was then performed to relieve bad contacts caused by unreasonable distances in the structure by keeping the protein and receptor restrained. The whole system was relaxed at 0 K with 10 Å non-bonded cutoff. The temperature of the system was then set to 300 K and equilibrated for 100 picoseconds with weak restraint on both receptor and protein, where bonds involving hydrogen are constrained using the SHAKE algorithm [22]. Torsion angles (Φ) were monitored throughout the simulation to determine the conformation of the glycosides. The Φ angle is defined as the angle between the O6-C2 bond of Sia and the glycosidic bond of Gal units (Figure 1). Note that the Φ angle defined by Xu and colleagues [15] refers to a different plane of rotation of the receptor. The Φ torsion angle described in this study was not considered in Xu *et al*. To determine the binding preference, the Φ angle was monitored in order to reflect the receptor type selectivity.

**Figure 2:**
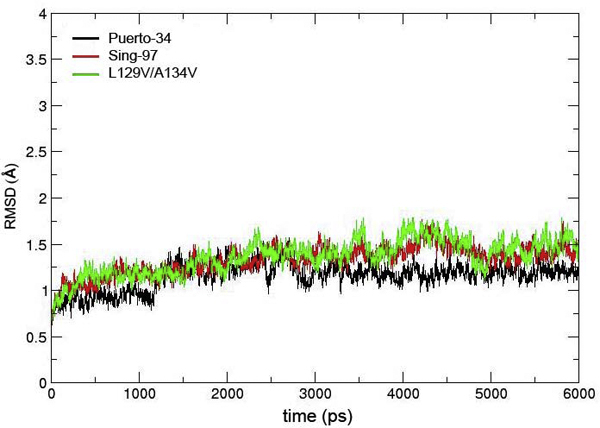
**Six nanosecond RMSD plots of three simulated HA systems**: Sing-97 (black), L129V/A143V Kan-1(red), and Puerto-34 (green).

The 3 nanosecond production run was performed at a constant temperature and pressure with 0.002 picoseconds time step (without restraining) using the SANDER module in the AMBER9 program [23]. The structures stabilized after 1.5 nanoseconds as shown in Figure 2. The highest degree of fluctuation was observed for residues located at the terminal chains of the structures. These residues were inserted in HA chain B and were left unrestrained during the simulations; however, they did not disturb the binding site (average root mean square deviation (RMSD) between residues 30 and 310 was less than 0.5 Å). The utility programs, Xmgrace [24], and VMD [25] were used to visualize and render all the figures presented in this paper.

## Results

Data from available co-crystal structures of cell receptor analog and HA were used to establish relationships between bound receptor conformation and host type preferences. Comparison between these structures revealed that the Φ torsion angle, has different values for Siaα(2,3)Gal and Siaα(2,6)Gal binding (Table 1). The observed values show Siaα(2,3)Gal has a Φ angle approximately -55 degrees in the H5 binding pocket, *i.e. *the receptor is in *trans *conformation. Meanwhile, Siaα(2,6)Gal exhibits a Φ angle of approximately +55 degrees, *i.e. *the receptor is in *cis *conformation [19]. In other words, Siaα(2,3)Gal seems to have an optimal binding geometry when the Gal and Sia units are bound in the *trans *conformation, while for the Siaα(2,6)Gal they are instead bound in the *cis *conformation. According to this, we hypothesized that in solution, both conformations are in equilibrium, as suggested by the available crystallographic data [5,16]. Upon binding of HA to the receptor, it is hypothesized that one conformation is favoured; thus, binding drives the equilibrium to this conformation without any molecular readjustment or thermodynamic cost.

In order to use the relationships between torsion angles and binding preferences to explain host selectivity of the unknown influenza virus structures, homology modelling and molecular dynamics (MD) simulations were employed. During each MD simulation, Φ was monitored and interpreted in terms of the binding preference.

**Figure 3:**
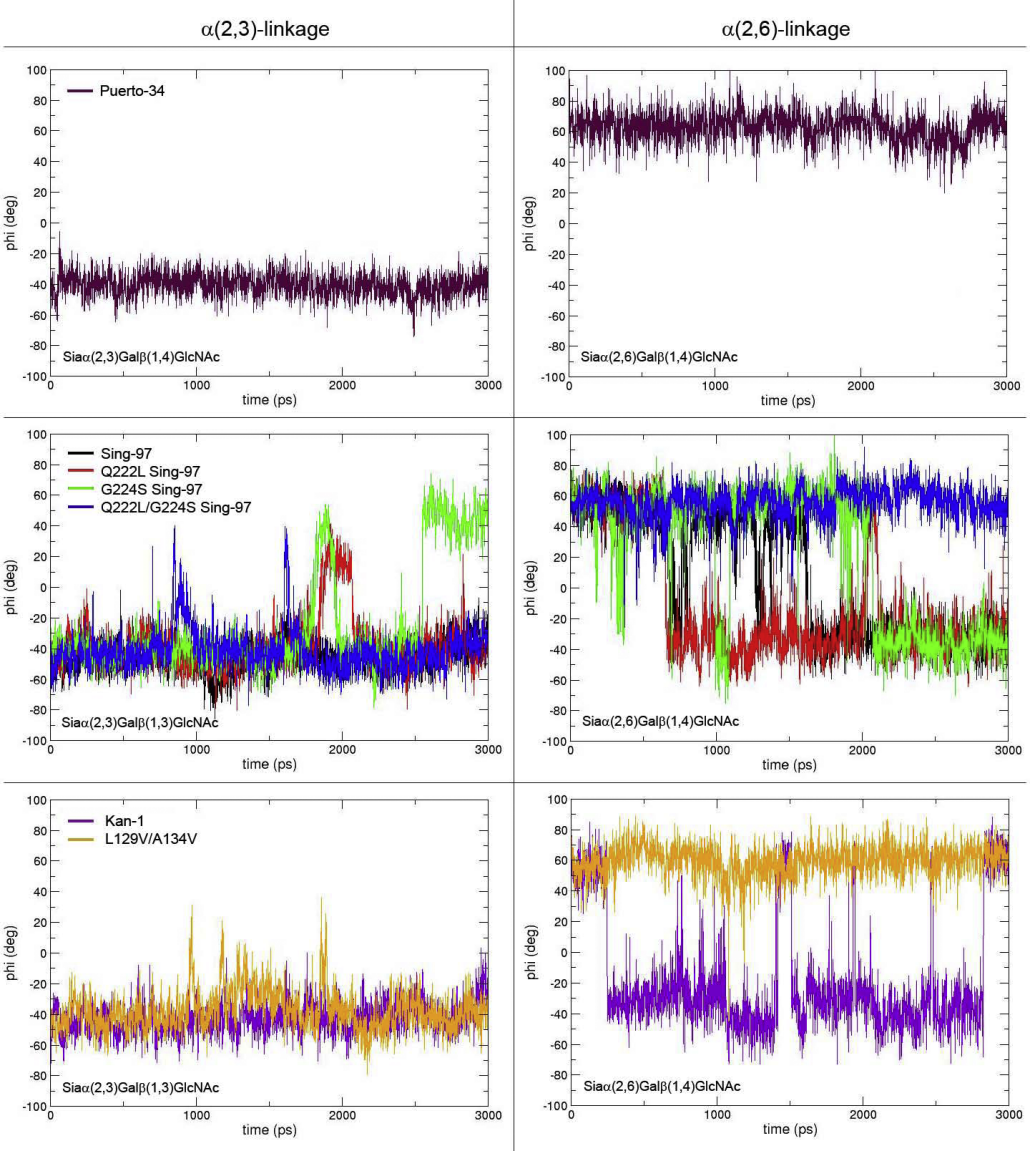
**Comparison of observed torsion angles (Φ) from the binding simulation between HA variants and avian- and human-type receptor analogs**. Receptor analogs in the left panel have a Siaα(2,3)Gal linkage (avian-type). Receptor analogs in the right panel have a Siaα(2,6)Gal linkage (human-type). Each panel demonstrates plots of torsion angles (Φ) in degrees recorded at the time intervals in picoseconds (ps) during MD simulations.

MD simulations were performed with different combinations of avian and human-type receptor analogs with HA variants. The duration of time in which the receptor analog spent in the *cis *or *trans *conformation when bound to each HA type varied according to the type of glycosidic linkage and the number of sugar units. Figure 3 presents plots showing different cis/trans conformations. For the binding studies of avian receptor analogs with α(2,3)-linkage, the glycosidic linkage in *trans *conformation was observed during most of the simulation in all HA systems tested (Φ angle -55 degrees), albeit with some transient fluctuations to the *cis *conformation for the Q222L, G224S, and Q222L/G224S Sing-97 HA variants.

In the simulation of human receptor analogs with α(2,6)-linkage, the *trans *configuration (Φ = -55 degrees) was observed in the majority during the simulation with some transient fluctuations to the *cis *conformation when bound to Sing-97, Sing-97 mutants Q222L, G224S and Kan-1 systems,. In contrast, the *cis *conformation was observed (Φ = +60 degrees) for the majority of the simulation time when the analogs bound with Puerto-34 human influenza, L129V/A134V Kan-1, and Q222L/G224S Sing-97 HAs.

## Discussion

From the experimental results, it can be concluded that each of the H5 HA variants exhibited different binding behaviours to different receptor analogs. The available X-ray structures contain only the tri-saccharide part of receptor analogs bound to the binding site, and the last two sugar units are missing or unsolved [5]. Nonetheless, the full-length receptor structures can be constructed using molecular modelling software [26-28]. In this study, modelling was restricted to the tri-saccharide receptor system to minimize the error in the simulations. In our previous work [19], Kan-1 was predicted to bind fairly well to human receptor for a significantly long period; however, this prediction is at variance with *in vitro *experimental data (Table 2). From the above experimental results, the tri-saccharide simulation model can better estimate the host type binding preference of different H5 HA variants than di-saccharide.

**Table 2:** MD predicted receptor type selectivity of HAs compared with data from published experimental assays.

Tested HA variants	Simulation		*in vitro*		
		
	α**2,3**	α**2,6**	α**2,3**	α**2,6**	Citation
***Puerto-34***	+++*	+++	NA	+++	[16]
Q222L Puerto-34	+++*	++	NA	NA	-
G224S Puerto-34	+*	+	NA	NA	-
Q222L/G224S Puerto-34	+++*	+++	NA	NA	-

***Sing-97***	+++	++	+++	+	[5]
Q222L Sing-97	++	++	++	++	[29]
G224S Sing-97	++	+	++	++	[29]
Q222L/G224S Sing-97	++	+++	+	+++	[29]

***Kan-1***	+++	+	+++	+	[19]
L129V/A134V Kan-1	+++	+++	+++	+++	[19]

NA - no data available, +++ most preferred, ++ moderately preferred, + least preferred

* - Siaα(2,3)Galβ(1,4)GlcNAc not Siaα(2,3)Galβ(1,3)GlcNAc

The crystallographic data in Ha *et al *[5] showed that the typical H5 HA binds preferentially to avian receptor with an α(2,3)-linkage in the *trans *conformation, whereas the typical H1 HA binds preferentially to human receptor with an α(2,6)-linkage in the *cis *conformation. The simulations presented here predicted that:

(i) Sing-97 and Kan-1 bound better to α(2,3) than to α(2,6), since the observed predominant conformation of receptor was *trans *for both receptor types

(ii) L129V/A134V Kan-1 could bind to both receptor types, since the *trans *conformation was predominant for α(2,3) binding and the *cis *conformation for α(2,6) binding

(iii) Q222L, G224S, Q222L/G224S Sing-97 mutants appear to have a weaker preference for α(2,3) than non-mutated Sing-97 because fluctuations from the *trans *to *cis *conformation were observed. The α(2,6) simulation of mutated Sing-97 implied that the Q222L/G224L variant had markedly greater binding affinity toward human cell receptor as it bound in *cis *with α(2,6) all the time.

(iv) Human virus Puerto-34 HA bound preferentially with α(2,6), since the observed conformation of receptor was in *cis *configuration.

### Prediction of the relative binding selectivity

Based on the HA binding conformation preferences from MD simulation (Table 2), predictions of relative binding selectivity (to host-type receptor) can be made as follows. The selectivity toward Siaα(2,3)Gal binding was similar among the three HA variants Puerto-34, Sing97 and Kan-1. The order of selectivity toward Siaα(2,6)Gal binding was Puerto-34 > L129V/A134V Kan-1 ≅ Q222L/G224S Sing-97 > Sing-97 HA ≅ Kan-1. These tendencies were in good agreement with the *in vitro *binding assays [18,19], in terms of order of preference. Therefore, the duration of the *cis *conformation during the simulation may be correlated with the selectivity of the docked HA.

**Figure 4:**
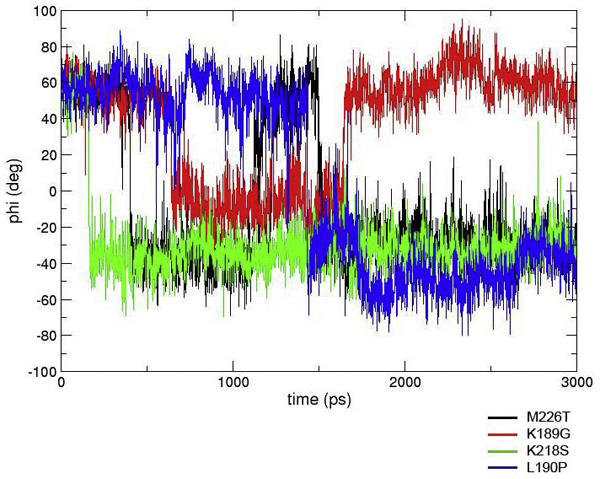
**Simulation results of four mutated-Kan-1 HA systems**. M226T, K189G, K218S and L190P binding to human receptor α(2,6) analog.

**Figure 5:**
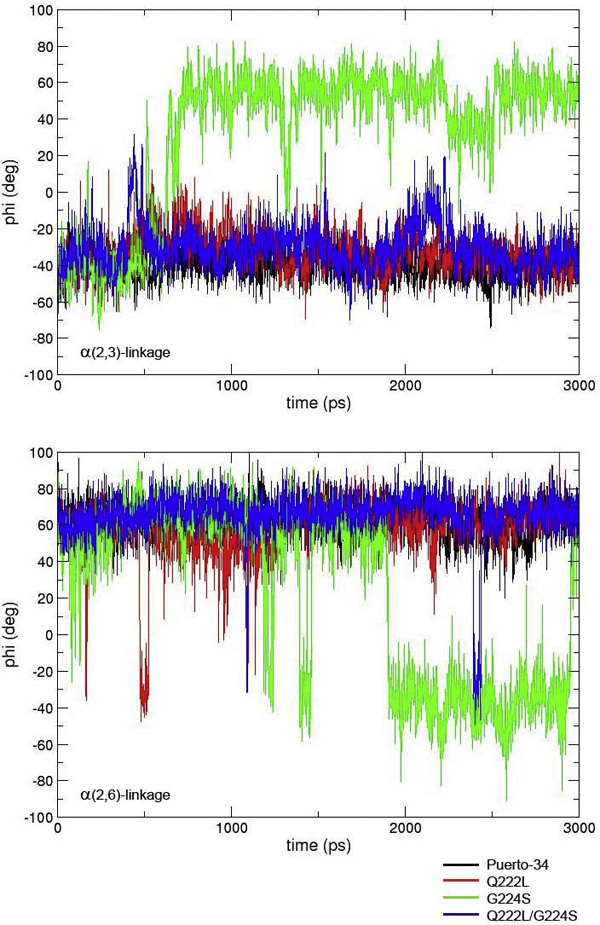
**Simulation results of four Puerto-34 HA systems**. non-mutated Puerto-34 (black), G224S (green), Q222L(red) and Q222L/G224S (blue) binding to α(2,3) receptor (top) and α(2,6) receptor (bottom).

According to our prediction scheme, the L129V/A134V Kan-1 variants, i.e. mutations M226T, K189G, K218S, and L190P, may have increased the selectivity slightly toward human receptor, since the receptor was present in *cis *conformation for some of the simulation (Figure 4). The results from the three mutated Puerto-34 systems also showed some changes in their binding behaviors compared to non-mutated Puerto-34 (Figure 5). The two single mutations, Q222L and G224S cause a loss in human receptor affinity as shown by fluctuations to the *trans*-conformation, while the double mutation Q222L/G224S maintained its preference for human receptor as the bound α(2,6) glycosides were in the *cis *conformation. For the α(2,3) receptor, all the Puerto-34 systems except for G224S appear to interact weakly since the receptor is in the *cis*-conformation. The *trans *conformation is observed for G224S, although the average torsion angle is increased from -50 to -30 degrees, suggesting that it may not be optimal for binding. The results show that mutations at residues 222 and 224 have minor impact on host preference for Puerto-34 HA

## Conclusion

We have shown that our *cis-trans *conformational analysis scheme could predict the host type selectivity of HA variants. Our *cis-trans *conformation hypothesis also worked well under another HA system where it predicted the *cis *conformation and revealed the similar mechanism in Puerto-34 simulation. The binding patterns and mechanisms of the adopted receptor model, Siaα(2,3)Gal and Siaα(2,6)Gal, to wild-type and mutated Kan-1 HA, and Sing-97 HA were proposed. The results could be used to explain why the L129V/A134V Kan-1 and Q222L/G224S Sing-97 could bind better to human receptor analog in *in vitro *assays. The underlying proposed mechanism that made H5 bind to human host without mutation at residue 222 or 224 involved the interaction between residue 134 side-chain and Gln222. It is proposed that mutations change the HA binding preference from Siaα(2,3)Gal to Siaα(2,6)Gal. Our study also suggested that even mutations outside of key binding residues [29], e.g. residue 222 or 224, have consequences on altering receptor type and should not be ignored. Furthermore, our procedure is useful for predicting host type, which can be tested by *in vitro *binding assays.

## Competing interests

The authors declare that they have no competing interests.

## Authors' contributions

NJ, DC and WM performed modeling, MD simulations and generated figures. ST, PJS and CS helped with data analysis and drafting of the manuscript. ST and CS obtained funding. All authors read and approved the final manuscript.

## Note

Other papers from the meeting have been published as part of *BMC Bioinformatics *Volume 10 Supplement 15, 2009: Eighth International Conference on Bioinformatics (InCoB2009): Bioinformatics, available online at http://www.biomedcentral.com/1471-2105/10?issue=S15.
